# Effectiveness of Different Intervention Modes in Lifestyle Intervention for the Prevention of Type 2 Diabetes and the Reversion to Normoglycemia in Adults With Prediabetes: Systematic Review and Meta-Analysis of Randomized Controlled Trials

**DOI:** 10.2196/63975

**Published:** 2025-01-29

**Authors:** Yachen Wang, Xin Chai, Yueqing Wang, Xuejun Yin, Xinying Huang, Qiuhong Gong, Juan Zhang, Ruitai Shao, Guangwei Li

**Affiliations:** 1 School of Population Medicine and Public Health Chinese Academy of Medical Sciences & Peking Union Medical College Beijing China; 2 The George Institute for Global Health University of New South Wales Newtown Australia; 3 Department of Neurology, Beijing Tiantan Hospital Chinese Academy of Medical Sciences & Peking Union Medical College Beijing China; 4 Fuwai Hospital Chinese Academy of Medical Sciences & Peking Union Medical College Beijing China

**Keywords:** mobile phone, prediabetic state, digital health intervention, intervention mode, lifestyle intervention, type 2 diabetes mellitus, meta-analysis, systematic review, review

## Abstract

**Background:**

Lifestyle interventions have been acknowledged as effective strategies for preventing type 2 diabetes mellitus (T2DM). However, the accessibility of conventional face-to-face interventions is often limited. Digital health intervention has been suggested as a potential solution to overcome the limitation. Despite this, there remains a significant gap in understanding the effectiveness of digital health for individuals with prediabetes, particularly in reducing T2DM incidence and reverting to normoglycemia.

**Objective:**

This study aimed to assess the effectiveness of different intervention modes of digital health, face-to-face, and blended interventions, particularly the benefits of digital health intervention, in reducing T2DM incidence and facilitating the reversion to normoglycemia in adults with prediabetes compared to the usual care.

**Methods:**

We conducted a comprehensive search in 9 electronic databases, namely MEDLINE, Embase, ACP Journal Club, Cochrane Central Register of Controlled Trials, Cochrane Database of Systematic Reviews, Cochrane Clinical Answers, Cochrane Methodology Register, Health Technology Assessment, and NHS Economic Evaluation Database through Ovid, from the inception to October 2024. This review included randomized controlled trials (RCTs) that studied the effectiveness of lifestyle interventions in adults with prediabetes. The overall intervention effect was synthesized using a random-effects model. The *I²* statistic was used to assess heterogeneity across the RCTs. We performed a subgroup analysis to explore the effectiveness of digital health, face-to-face, and blended interventions compared with the control group, which received usual care.

**Results:**

From an initial 7868 records retrieved from 9 databases, we identified 54 articles from 31 RCTs. Our analysis showed that face-to-face interventions demonstrated a significant 46% risk reduction in T2DM incidence (risk ratio [RR] 0.54, 95% CI 0.47-0.63; *I*²=43%; *P*<.001), and a 46% increase in the reversion to normoglycemia (RR 1.46, 95% CI 1.11-1.91; *I*²=82%; *P*=.006), when compared with the control group. On the other hand, digital health interventions, compared with the control group, were associated with a 12% risk reduction in T2DM incidence (RR 0.88, 95% CI 0.77-1.01; *I*²=0.6%; *P*=.06). Moreover, the blended interventions combining digital and face-to-face interventions suggested a 37% risk reduction in T2DM incidence (RR 0.63, 95% CI 0.49-0.81;*I*²<0.01%; *P*<.001) and an 87% increase in the reversion to normoglycemia (RR 1.87, 95% CI 1.30-2.69; *I*²=23%; *P*=.001). However, no significant effect on the reversal of prediabetes to normoglycemia was observed from the digital health interventions.

**Conclusions:**

Face-to-face interventions have consistently demonstrated promising effectiveness in both reductions in T2DM incidence and reversion to normoglycemia in adults with prediabetes. However, the effectiveness of digital health interventions in these areas has not been sufficiently proven. Given these results, further research is required to provide more definitive evidence of digital health and blended interventions in T2DM prevention in the future.

**Trial Registration:**

PROSPERO CRD42023414313; https://tinyurl.com/55ac4j4n

## Introduction

Type 2 diabetes mellitus (T2DM) is a significant global public health issue. Up to 2021, there were 529 million individuals with T2DM [1], and 6.7 million deaths attributable to T2DM [2] with a cost of over a trillion dollars in health care worldwide [3].

Prediabetes is a high-risk condition for developing T2DM [4]. Without appropriate interventions, 5% to 18% of prediabetes will progress to T2DM annually [5]. In 2018, the estimated prevalence of prediabetes among Chinese adults was 38%, highlighting the urgent need for early interventions to prevent the progression to T2DM.

According to the World Health Organization (WHO) definition provided in 1998, a lifestyle intervention aims to modify an individual’s way of living and improve their physical and psychological health by changing patterns of behavior that are harmful to health [6]. There is substantial evidence indicating that lifestyle interventions can prevent T2DM progression [7-9], and even reverse prediabetes to normoglycemia [10,11]. However, most of this evidence stems from face-to-face interventions, which are time-consuming and reach a limited audience. Digital health intervention, defined as a discrete functionality of digital technology, is applied to achieve health objectives and is implemented within digital health applications, including SMS, smartphones, and wearable devices, to achieve health objectives. Meanwhile, it offers immense opportunities for self-management and the prevention of chronic diseases [12-14].

While previous meta-analyses have demonstrated the effectiveness of digital health interventions in promoting physical activity and dietary changes, leading to weight loss and improved glucose control in individuals with prediabetes [15-17], evidence regarding their effectiveness in reducing T2DM incidence and reverting prediabetes to normoglycemia remains limited. Therefore, T2DM incidence and reversion to normoglycemia are essential endpoints for providing evidence on the application of digital health interventions in T2DM prevention.

This systematic review and meta-analysis aims to evaluate the effectiveness of face-to-face, digital health, and blended interventions in reducing T2DM incidence and reversing prediabetes to normoglycemia based on randomized controlled trials (RCTs).

## Methods

### Overview

The systematic review followed the PRISMA (Preferred Reporting Items for Systematic Review and Meta-Analysis) 2020 checklist [18]. The review protocol was registered at PROSPERO (International Prospective Register of Systematic Reviews; CRD42023414313).

### Search Strategy and Eligible Criteria

Electronic searches of 9 databases (MEDLINE, Embase, ACP Journal Club, Cochrane Central Register of Controlled Trials, Cochrane Database of Systematic Reviews, Cochrane Clinical Answers, Cochrane Methodology Register, Health Technology Assessment, and NHS Economic Evaluation Database) were done through Ovid. We performed the initial search on May 31, 2022, and updated the search on October 2, 2024, with no language restrictions. The search terms were developed in consultation with librarians incorporating MeSH (Medical Subject Headings) terms and text words and piloted before being used in the current review. The search strategy was: “prediabetes or high-risk of diabetes” and “lifestyle intervention or digital health intervention” and “RCTs.” The detailed search strategy can be found in Multimedia Appendix 1 [15,19]. The reference lists of the included studies and previous reviews were thoroughly hand-searched to identify any additional relevant research. The eligibility criteria were guided by the Population, Intervention, Comparison, Outcome, and Study Design (PICOS) framework, as illustrated in Textbox 1.

**Population, Intervention, Comparison, Outcome, and Study Design (PICOS) to determine the eligibility**

**Inclusion criteria**

- Population
- Prediabetes, including impaired fasting glucose, impaired glucose tolerance, impaired fasting glucose and impaired glucose tolerance, elevated hemoglobin A_1c_, and intermediate hyperglycemia by different guidelines (Multimedia Appendix 2 [20-25])
- Intervention
- Lifestyle intervention (see “Definition and Examples of Lifestyle Intervention”) lasted for at least 1 year.
- Comparison
- Usual care.
- Outcome
- Type 2 diabetes mellitus incidence or reversion to normoglycemia.
- Study design
- Randomized controlled trial.

**Exclusion criteria**

- Population
- High risk of diabetes identified by risk scores.
- Intervention
- Pharmacological intervention or a combination of pharmacological and lifestyle intervention.
- Comparison
- Pharmacological intervention.
- Outcome
- Intermediate outcomes such as weight loss.
- Study design
- Nonrandomized controlled trial.

A total of 2 authors (YCW and XC) independently screened the titles and abstracts of studies and assessed the full text for eligibility. Disagreements, if any, were resolved through discussion with a third author (XJY). For efficient management of the selection process, all retrieved studies were imported into Covidence.

### Definition and Examples of Lifestyle Intervention

According to the 1998 World Health Organization definition, a lifestyle intervention aims to modify an individual’s way of living and improve their physical and psychological health by changing patterns of behavior that are harmful to health [6].

For example, in our meta-analysis, the behavior pattern changes mainly include nutrition, physical activity, and weight management.

### Data Extraction and Quality Assessment

Data extraction was carried out independently using standard data extraction forms. Extracted data included (1) study information (author, year of publication, and study location); (2) study participants (prediabetes phenotype, diagnostic criteria, ethics, age, the proportion of male participants, baseline, BMI, fasting plasma glucose [FPG], and sample size); (3) intervention and control details (the intervention treatment, the control group, intervention settings, delivery format, intervention mode, intervention duration, and the follow-up duration was defined from the intervention year to the first year in which reported T2DM incidence occurred, as well as the time to reversion to normoglycemia); (4) for the studies applying digital health interventions, the theoretical framework, intervention adherence, digital health intervention categories, and its intervention components categorized by the WHO [26]; (5) outcome measurements; and (6) key study results including T2DM events, proportion or numbers of individuals reverting to normoglycemia and effect size between intervention group and control group.

The risk of bias was assessed using the revised Cochrane Risk-of-Bias tool (RoB 2.0) for randomized trials [27]. The assessment considered the following elements: (1) randomization process, (2) deviations from intended interventions, (3) missing outcome data, (4) measurement of the outcome, (5) selection of the reported result, and (6) overall bias.

Data extraction and quality assessment were independently carried out by 2 authors (YCW and XC). Any disagreements between the 2 authors were resolved through discussion or with a third author (XJY).

### Synthesis of Results

Results were sought for both baseline and end-of-intervention or postintervention outcome data, specifically focusing on the data that first observed the primary outcome. If the original article provided 95% CI or SE without SD, we converted them into SD, following the guidelines in the Cochrane Handbook for Systematic Reviews of Interventions [27]. To facilitate the interpretation of the findings, we used standardization techniques to unify measurement units across studies. Both blood glucose and blood lipids indicators were measured in mmol/L, except hemoglobin A_1c_ (HbA_1c_) in percentage.

The primary outcomes of this systematic review and meta-analysis were the incidence of T2DM and the reversion from prediabetes to normoglycemia, both compared with the usual care, expressed in terms of risk ratios (RRs), hazard ratios, or odds ratios. Secondary outcomes encompassed the changes in blood glucose, specifically FPG, 2-hour postprandial glucose, and HbA_1c_. This study also examined variations in key indicators including body weight, BMI, waist circumference, blood pressure, and blood lipid profiles. The lipid profiles encompass triglycerides, high-density lipoprotein cholesterol, low-density lipoprotein cholesterol, and total cholesterol.

### Statistical Analysis

Intervention effects were defined as the differences in outcomes between the intervention and control groups. For dichotomized outcomes, synthesized measures were presented as RRs; and for continuous outcomes, we presented mean differences. The pooled estimates were synthesized by random-effect models and presented in the forest plots. The *I*² statistic was used to assess statistical heterogeneity between trials. For studies with 2 or more intervention groups, each group was treated independently and annotated in the forest plots [28].

A prespecified subgroup analysis was conducted to assess the effectiveness of digital health interventions, face-to-face lifestyle interventions, and the combined application compared with the usual care. The subgroups were categorized into 3 intervention modes [29], face-to-face, digital health, and blended. Based on the provision of behavioral support, digital health interventions can be divided into stand-alone interventions and digital support interventions (Textbox 2). Sensitivity analysis was conducted to verify the robustness of the pooled RRs. Funnel plot and Egger test were used to investigate the publication bias [30]. All analyses were carried out using Stata (version 16.0; StataCorp). We considered *P* less than .05 as significant.

**Categories of examples of intervention modes based on the provision of behavioral supports.**

(i) Face-to-face interventions

- In-person support from a counselor face-to-face: for example, group sessions on lifestyle modification.

(ii) Digital health interventions

- Stand-alone interventions: participants were not offered ongoing support from a counselor. For example, self-management or self-monitor smartphone apps.
- Digital support intervention: remote support with behavioral support through mobile health technologies. For example, emails, text messaging prompts, or phone calls with a counselor.

(iv) Blended interventions

- Combination of face-to-face intervention and digital health intervention.

### Ethical Considerations

This review does not involve human subject information, primary data collection, or any form of experimentation on individuals.

## Results

### Study Selection

**Figure 1:**
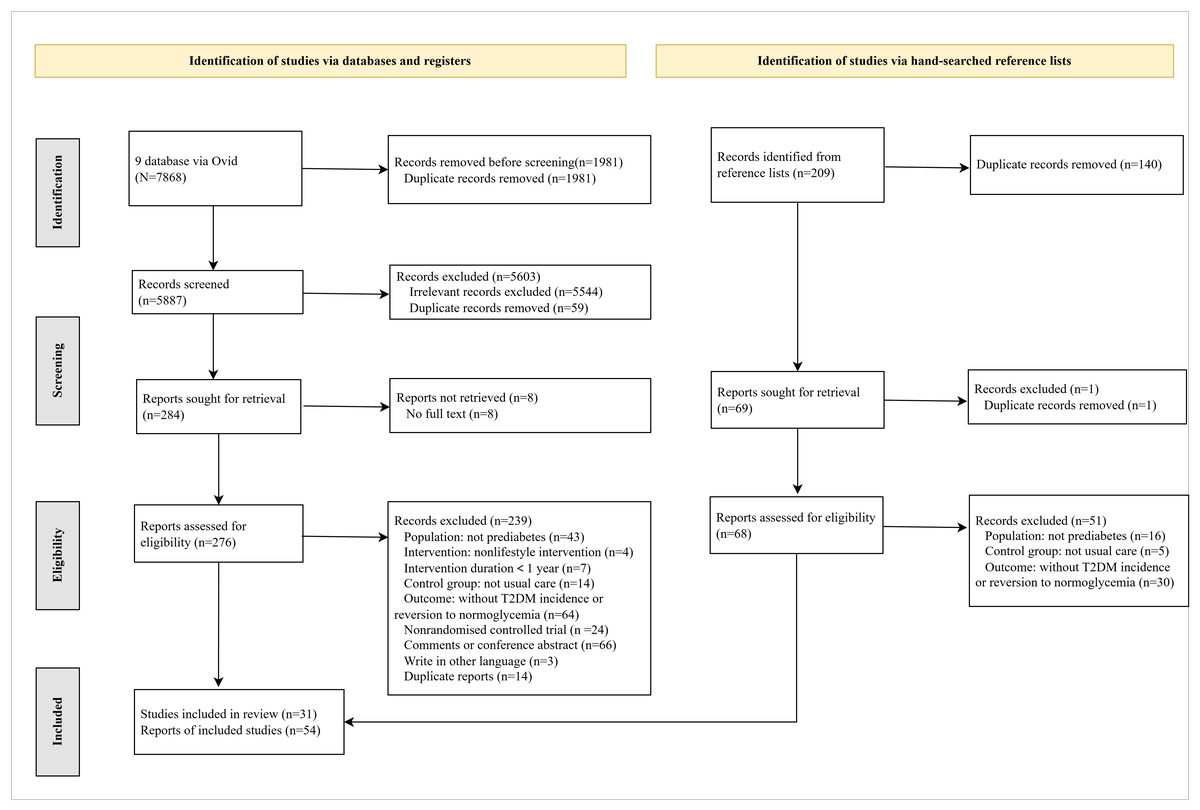
PRISMA (Preferred Reporting Items for Systematic Review and Meta-Analysis) flow diagram describing the literature review process. T2DM: type 2 diabetes mellitus.

The database search yielded 7868 articles from the 9 databases and identified 37 articles as eligible. We manually identified an additional 209 articles from the reference list of relevant reviews, of which 17 articles were deemed eligible. Finally, a total of 54 articles from 31 studies met the eligibility criteria and were included in the review (Figure 1).

### Study Characteristics

Baseline characteristics of the included studies are shown in Multimedia Appendix 3 [7-11,31-56]. The systematic review and meta-analysis included 23,684 individuals with prediabetes in total, with studies ranging from 48 to 2865 participants. Prediabetes identifications were primarily based on the WHO criteria since 1999 (15/31, 48%), which defined prediabetes by FPG of 6.1 to 6.9 mmol/L or 2-hour postprandial glucose of 7.8 to 11.0 mmol/L (Multimedia Appendix 2 [20-25]). Among the included studies, the majority (18/31, 58%) reported an average participant age exceeding 50 years. Among 31 studies, 30 studies reported the T2DM incidence as the outcome [7-11,31-54,57], and 10 studies reported the reversion to normoglycemia as the outcome [8-11,36,38,39,44,53,55].

### Description of Interventions

Intervention characteristics are outlined in Multimedia Appendix 3 [7-11,31-56], including intervention content, comparative interventions, settings, delivery method, intervention duration, and follow-up duration. Intervention durations and follow-up durations across the studies varied, ranging from 1 to 6 years. A majority of the studies (22/31, 70% [7-10,35-38,40-44,46-52,55,57]) used face-to-face intervention, 6 studies used digital health intervention [10,11,32,34,45,54], and 6 studies used blended intervention [31,33,39,50,53,57]. No study with digital health intervention used stand-alone intervention. In the face-to-face interventions, group sessions (22/28, 79%) were the most common delivery format applied (Multimedia Appendix 3 [7-11,31-56]).

Of all the studies that included digital health components, SMS text messaging (5/12, 42%) and telephone contacts (6/12, 50%) were the most used. One of these studies used a web-based approach [11], and 3 studies used wearable technology [11,31,58]. According to the WHO classification of digital health interventions, targeted client communication was the most used digital health category (11/12, 92%), followed by personal health tracking (4/12, 33%). Transmitting targeted health information to clients was the intervention mostly used in targeted client communication including behavior change communication, health promotion communication, and client-centered SMS text messaging. Among the 12 studies that included digital health interventions, only 5 were published within the last 5 years [10,11,45,50,53]. The transtheoretical model of behavioral change and social cognitive theory was the most common theoretical method used in digital health interventions.

### Main Outcomes

#### Type 2 Diabetes Mellitus Incidence

**Figure 2:**
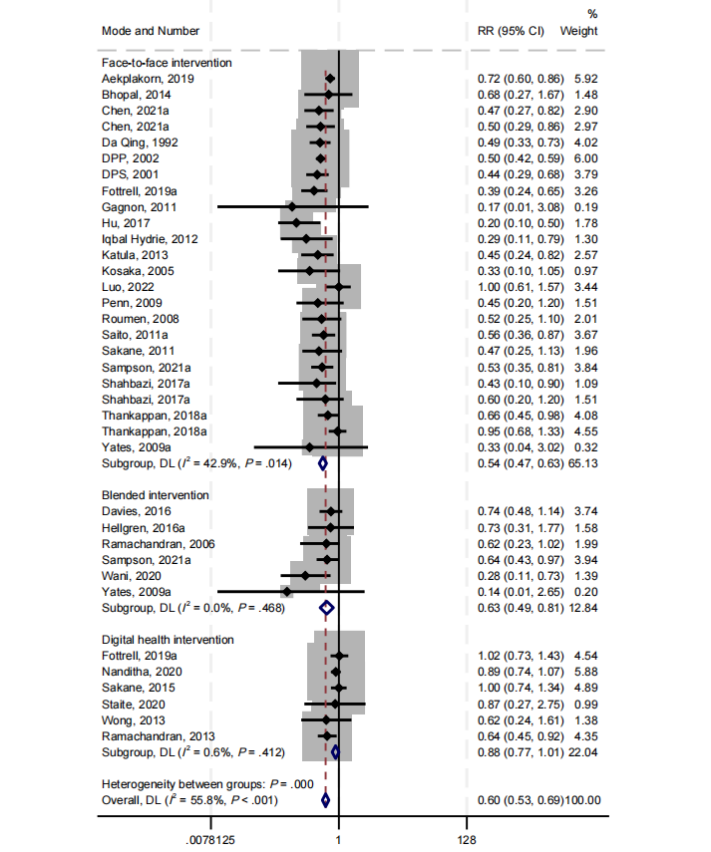
The forest plot on the reduction of type 2 diabetes mellitus incidence by intervention mode subgroups (face-to-face, blended, and digital health interventions) compared with the usual care group [7-11,26-30,51-70]. Multiple intervention arms within a single study were treated as distinct intervention groups. DL: DerSimonian-Laird, a method to estimate the heterogeneity variance.

The pooled estimate of 30 studies revealed the significant effectiveness of lifestyle interventions in reducing the incidence of T2DM (RR 0.60, 95% CI 0.53-0.69; *I^2^*=55%; *P*<.001; Figure 2).

In subgroup analysis, face-to-face interventions were found to reduce the incidence of T2DM among adults with prediabetes by 46% (RR 0.54, 95% CI 0.47-0.63; *I^2^*=43%; *P*<.001) compared with the control group (Figure 2). The digital health interventions (RR 0.88, 95% CI 0.77-1.01; *I^2^*=0.6%; *P*=.06) observed a 12% risk reduction in T2DM incidence compared with the control group, although no statistical difference was observed. Notably, one study reported a 36% risk reduction in T2DM incidence through SMS test messaging [32]. However, no statistically significant difference was observed in the other two similar studies, despite observing a reduction in T2DM incidence [33,57]. Studies using telephone contacts and voice messages did not report any significant reduction in T2DM incidence [10,34]. The blended interventions (RR 0.63, 95% CI 0.49-0.81; *I^2^*＜0.01%; *P*<.001) observed a 37% risk reduction in T2DM incidence compared with the control group.

#### Reversion From Prediabetes to Normoglycemia

**Figure 3:**
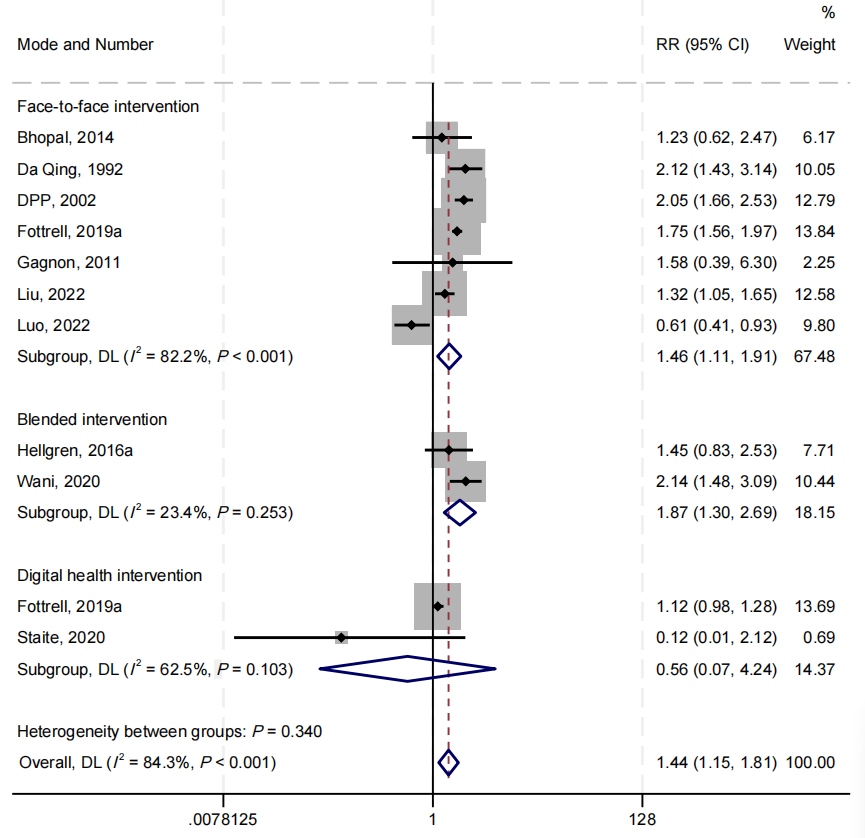
The forest plot on reversion to normoglycemia by intervention mode subgroups (face-to-face, blended, and digital health interventions) compared with the usual care group [8-11,52,54,55,60,69,71]. Multiple intervention arms within a single study were treated as distinct intervention groups. DL: DerSimonian-Laird, a method to estimate the heterogeneity variance.

A total of 10 studies were included in the meta-analysis of reversion from prediabetes to normoglycemia [8-11,36,38,39,44,53,55]. Compared with the control group, the lifestyle intervention group demonstrated a 44% increase in the rate of reversion from prediabetes to normoglycemia (RR 1.44, 95% CI 1.15-1.81; *I*^2^=84%; *P*<.001; Figure 3).

In subgroup analysis (Figure 3), the face-to-face intervention and blended intervention group exhibited significant effectiveness by 46% (RR 1.46, 95% CI 1.11-1.91; *I^2^*=82%; *P*=.006) and 87% (RR 1.87, 95% CI 1.30-2.69; *I^2^*=23%; *P*=.001) increase in reversion to normoglycemia compared with the control group, respectively. A total of 2 studies used digital health interventions: one used voice messages [10], while the other incorporated wearable technology, web-based education, and motivational SMS text messages [11]. In the digital health intervention group, neither of the 2 studies observed significant effectiveness of digital health interventions on the reversion of prediabetes to normoglycemia. One study [10] observed the potential effectiveness on reversion but produced no statistical difference (RR 1.12, 95% CI 0.98-1.28) [10]. In another study [11], the CI was too wide to determine the effect (RR 0.12, 95% CI 0.01-2.12).

#### Secondary Outcomes

Face-to-face interventions’ standard mean difference (SMD=–0.25, 95% CI –0.35 to –0.15; *I*^2^=48%; *P*<.001) demonstrated a significant decrease in weight loss than digital health interventions (SMD=–0.07, 95% CI –0.17 to 0.03; *I^2^*=48%; *P*=.15). Similarly, reductions in waist circumference, BMI, FPG, 2-hour postprandial glucose, HbA_1c_, systolic blood pressure, diastolic blood pressure, and TG were more effectively pronounced in the face-to-face intervention. Contrarily, low-density lipoprotein cholesterol showed a significant decrease through digital health interventions (SMD=–0.10, 95% CI –0.19 to –0.02; *I^2^*=0%; *P*=0.01) while no effectiveness was observed in face-to-face interventions (SMD=–0.13, 95% CI –0.41 to 0.14; *I^2^*=81%; *P*=.34; Multimedia Appendix 4).

### Risk-of-Bias Assessment and Publication Bias

Among 30 studies that reported T2DM incidence as the outcome, 10 studies were classified as “low risk” [9,10,32,36,42,44,45,48,52,56], 7 studies as having “some concerns” [7,8,31,33,39,40,54], and the remaining 13 studies as “high risk” [11,34,35,37,38,41,43,46,47,49-51,53], assessed by RoB 2.0 judgment. Among the 10 studies that reported reversion to normoglycemia as the outcome, 4 studies were classified as “low risk” [9,10,36,44], 2 studies as having “some concerns” [8,39], and the remaining 4 studies as “high risk” [11,38,53,55] (Multimedia Appendix 4). The high risk of bias primarily stemmed from the biased selection of reported results, followed by missing outcome data. In other domains, most studies were considered low risk. Publication bias was detected for T2DM incidence (Egger test *P*=.02), but not for prediabetes reversion (Egger test *P*=.66). Funnel plots suggested that studies with small sample sizes were less likely to be published (Multimedia Appendix 4).

### Sensitivity Analysis

The sensitivity analysis found no significant change after excluding studies one by one in both T2DM prevention and reversion to normoglycemia. After excluding studies with certain concerns and those deemed to be at high risk of bias, sensitivity analysis indicates that our meta-analysis results for T2DM incidence are stable. However, the number of studies on reversion to normoglycemia was too small to conduct a meta-analysis (Multimedia Appendix 4).

## Discussion

### Principal Findings

Our systematic review and meta-analysis demonstrated that face-to-face and blended interventions can effectively prevent T2DM incidence and promote reversion to normoglycemia. Digital health interventions would be a possible solution in T2DM prevention based on our review; however, more evidence is required to substantiate the effectiveness. There is limited evidence to support digital health intervention in reversion to normoglycemia among adults with prediabetes.

The findings in face-to-face interventions showed consistent effectiveness in T2DM prevention and reversion to normoglycemia, which is similar to the previous meta-analysis reported [59,60]. In addition, the study found that the face-to-face intervention group showed greater improvements in blood glucose, blood lipid levels, and other indicators compared with the digital health group, which is similar to previous studies [15].

The results of the blended interventions, which combine face-to-face and digital health interventions, showed significant effectiveness in reducing T2DM incidence and promoting the reversion to normoglycemia. This is in accordance with our earlier observations, which showed that blended intervention can effectively help people lose weight [61].

Although no statistical significance is observed in digital health intervention, the risk reduction in T2DM incidence indicates its potential effectiveness. These results are similar to those reported by previous systematic reviews among older adults with prediabetes including 3 RCTs [62], which contrast with earlier studies that reported the effectiveness of glucose indicators or lifestyle modification [63] in digital health intervention.

The inconsistency between studies using T2DM incidence and those focusing on weight loss and lifestyle changes as outcomes in digital health interventions may stem from several factors. First, it necessitates a multiyear observation period to ascertain the effectiveness of these interventions on the T2DM incidence and the reversal of hyperglycemia [7-9]. However, the studies of digital health intervention included in this meta-analysis had relatively short intervention periods ranging from 1 to 3.8 years, which may not be sufficient for observing the effectiveness. Second, it is recognized that adherence is an important indicator of expected good results [15,32]; however, 10 of 12 studies with digital health intervention included in this meta-analysis have not provided adherence information, which may explain why the digital health intervention has not reported the significant changes [10,11,31,32,39,45,50,53,54,56]. Finally, a possible explanation for the results may be the lack of adequate studies, resulting in CIs that are too wide to detect significance. Therefore, we should interpret the results with caution in digital health interventions.

### Implications

Face-to-face lifestyle intervention, even when effective, adherence is not ideal in the real world [64]. Recently, studies have used digital health intervention in the Diabetes Prevention Program (DPP) in the real world, called the digital DPP [65,66]. The results showed that, in comparison to traditional face-to-face small-group education, the dDPP exhibits good reach and adherence [67]. Also, promising effectiveness in weight loss was found in dDPP; however, it is unclear in the T2DM incidence in the real world [65,68]. It is recognized that behavioral changes necessitated by such interventions require a more extended time period to assess their impact on T2DM incidence [69,70], although the American Diabetes Association recommends using digital health interventions in diabetes prevention programs for its effectiveness in glycemic control [71]. Therefore, more studies are needed to explore the effectiveness of digital health and blended interventions, observing outcomes related to T2DM incidence and reversion to normoglycemia, in order to provide more evidence for T2DM prevention.

Moreover, a prevailing challenge associated with digital health interventions is the observed decline in adherence over extended periods of use, which may account for the generally short intervention durations characteristic of digital health interventions [65]. Thus, digital health interventions should use certain behavioral change techniques or adhere to a theoretical model, as demonstrated in previous studies [72-74], as most studies used the transtheoretical model of behavioral change and social cognitive theory in lifestyle modification. Future research should not only focus on lifestyle modification but also collect information on adherence using digital health interventions.

In addition, for high-risk patients, the dDPP group had, on average, lower costs [75]. For social care use, cost-effectiveness analyses also support the preferential selection of DPPs over face-to-face small-group education per quality-adjusted life years [68]. Further research is required to develop accessible, effective, and cost-efficient diabetes prevention interventions incorporating digital health.

Furthermore, although the studies included in our meta-analysis did not use machine learning or generative artificial intelligence, these technologies have now been extensively applied in chronic disease prevention and control. In the future, machine learning with data from wearable devices will be capable of making informed judgments and providing individualized recommendations for the precision prevention and treatment of diabetes through the application of generative artificial intelligence [76]. However, it is crucial to pay attention to its fidelity to maximize its value and promote health care equity, given the challenges of compatibility between different artificial intelligence devices, standardization of data, and risks to data security [77,78].

Finally, even though there are several digital health interventions, digital support interventions have been used in diabetes prevention research limited to T2DM incidence and normoglycemia for the primary aim. For instance, a recent meta-analysis of diabetes management found that smartphone app and SMS test message interventions, but not website-based interventions, were associated with better glycemic control [15], which was defined as the primary aim of the research. Once the results with T2DM incidence and reversion of normoglycemia are available sufficiently, analyses can be performed to further explore the effective components within digital health interventions.

### Strengths and Limitations

To the best of our knowledge, this is the first meta-analysis to assess the effectiveness of digital health and blended interventions on T2DM prevention using T2DM incidence and reversion to normoglycemia instead of weight loss and changes in blood glucose levels. We conducted a quantitative synthesis to explore the effectiveness of digital health interventions in T2DM prevention, which is different from other systematic reviews constrained by a limited number of studies to conduct a meta-analysis.

The study also has some limitations. First, digital health has been widely used in health research; however, 5 of 12 studies encompassed digital health interventions were published within the last 5 years, which does not meet the expectation in novel studies. To ensure robust findings, we conducted sensitivity analyses to exclude studies with potential biases from small samples or weak designs. Second, publication bias was found in the study for the T2DM incidence, and these results need to be interpreted with caution. Third, the limited studies on digital health interventions restrict our ability to conduct subgroup analyses on the effectiveness of their components. We have summarized the components and their theoretical bases in our results to aid further research. Finally, our research does not directly compare the effectiveness of different intervention modes; instead, it focuses on comparisons within control groups due to the lack of direct comparative studies in the literature.

### Conclusion

While digital health interventions for lifestyle changes suggest potential effectiveness in preventing T2DM, further evidence is needed to confirm their effectiveness. There is limited evidence supporting the effectiveness of digital health interventions in reversing prediabetes. Face-to-face lifestyle interventions are effective but limited by accessibility and low adherence. With the rapid annual increase of diabetes globally, there is an urgent need to prevent T2DM, particularly among those with prediabetes. Future research is needed to consolidate the effectiveness of interventions incorporating digital health in T2DM prevention and to increase the accessibility of lifestyle interventions.

## Appendix

Multimedia Appendix 1 Search strategy.

Multimedia Appendix 2 Diagnosis and classification of prediabetes in different guidelines.

Multimedia Appendix 3 Characteristics of the included studies.

Multimedia Appendix 4 Assessment of secondary outcomes, evaluation of publication bias, and sensitivity analysis.

Multimedia Appendix 5 PRISMA checklist.

## Abbreviations

DPP: Diabetes Prevention Program
FPG: fasting plasma glucose
HbA1c: hemoglobin A1c
MeSH: Medical Subject Headings
PICOS: Population, Intervention, Comparison, Outcome, and Study design
PRISMA: Preferred Reporting Items for Systematic Review and Meta-Analysis
PROSPERO: International Prospective Register of Systematic Reviews
RCT: randomized controlled trial
RoB 2.0: Cochrane Risk-of-Bias tool
RR: risk ratio
SMD: standard mean difference
T2DM: type 2 diabetes mellitus
WHO: World Health Organization

## Data Availability

The datasets generated during and/or analyzed during this study are available from the corresponding author on reasonable request.
