# Supplementary material for: Effectiveness of Different Intervention Modes in Lifestyle Intervention for the Prevention of Type 2 Diabetes and the Reversion to Normoglycemia in Adults With Prediabetes: Systematic Review and Meta-Analysis of Randomized Controlled Trials
Source: J Med Internet Res. 2025 Jan 29;27:e63975. doi: 10.2196/63975 (PMC11822313; doi:10.2196/63975)
Supplement: Multimedia Appendix 1 [file jmir_v27i1e63975_app1.docx]

**Search strategy**

EBM Reviews - ACP Journal Club

EBM Reviews - Cochrane Central Register of Controlled Trials

EBM Reviews - Cochrane Database of Systematic Reviews

EBM Reviews - Cochrane Clinical Answers

EBM Reviews - Cochrane Methodology Register

EBM Reviews - Health Technology Assessment

EBM Reviews - NHS Economic Evaluation Database

Embase

Ovid MEDLINE(R)

| Number | Search strategy |
| --- | --- |
| 1 | exp Prediabetic State/ |
| 2 | exp Hyperglycemia/ |
| 3 | ((impaired fasting adj2 glucose) or IFG or impaired FPG).ab,ti. |
| 4 | ((impaired glucose adj (tolerance or metabolism)) or IGT).ab,ti. |
| 5 | (impaired glucose regul* or IGR or fasting plasmas glucose or glucose*intolerance or raised glucose or non - diabetic hyperglycaemia or "NDH" or "intermediate hyperglyc?emi*" or impaired glycaemia).ab,ti. |
| 6 | (impaired glucose stat* or impaired glucose respons* or impaired glucose control* or impaired glucose homeost* or reduced glucose metab*).ab,ti. |
| 7 | (pre?diabet* or borderline diabet* or mild diabet*).ab,ti. |
| 8 | (("HbA(1c)" or HbA1 or HbA1c or "HbA 1c" or ((glycosylated or glycated) adj h?emoglobin)) adj (abnormal or raised or elevated or high or baseline)).ab,ti. |
| 9 | (diabetes prevention adj (program* or stud* or trial?)).ab,ti. |
| 10 | (risk adj2 ("type 2" or "type II" or diabetes or T2D* or NIDDM)).ab,ti. |
| 11 | 1 or 2 or 3 or 4 or 5 or 6 or 7 or 8 or 9 or 10 |
| 12 | exp Life Style/ |
| 13 | risk reduction behavior/ |
| 14 | diet, diabetic/ or diet, fat-restricted/ or diet, mediterranean/ or diet, reducing/ or diet, healthy/ |
| 15 | Exercise/ |
| 16 | ((lifestyle or life style) adj3 (intervention? or change* or modif* or program or programme)).ab,ti. |
| 17 | (nutrition* adj3 (intervention? or change* or modif* or program or programme)).ab,ti. |
| 18 | mhealth.mp. or exp Mobile Health/ |
| 19 | telehealth.mp. or exp Telemedicine/ |
| 20 | exp Digital Interventions/ or exp Computer Applications/ or exp Internet/ or digital health.mp. or exp Distance Education/ or exp Electronic Health Services/ or exp Telemedicine/ or exp Technology/ or exp Digital Divide/ |
| 21 | exp Internet/ or exp Online Therapy/ or online health.mp. |
| 22 | teledietetics.mp. or exp Telemedicine/ |
| 23 | "randomized controlled trial".pt. |
| 24 | (random$ or placebo$ or single blind$ or double blind$ or triple blind$).ti,ab. |
| 25 | (retraction of publication or retracted publication).pt. |
| 26 | (animals not humans).sh. |
| 27 | ((comment or editorial or meta-analysis or practice-guideline or review or letter) not "randomized controlled trial").pt. |
| 28 | (random sampl$ or random digit$ or random effect$ or random survey or random regression).ti,ab. not "randomized controlled trial".pt. |
| 29 | 23 or 24 or 25 |
| 30 | 26 or 27 or 28 |
| 31 | 29 not 30 |
| 32 | 12 or 13 or 14 or 15 or 16 or 17 or 18 or 19 or 20 or 21 or 22 |
| 33 | 11 and 31 and 32 |
