# Supplementary material for: Effectiveness of Different Intervention Modes in Lifestyle Intervention for the Prevention of Type 2 Diabetes and the Reversion to Normoglycemia in Adults With Prediabetes: Systematic Review and Meta-Analysis of Randomized Controlled Trials
Source: J Med Internet Res. 2025 Jan 29;27:e63975. doi: 10.2196/63975 (PMC11822313; doi:10.2196/63975)
Supplement: Multimedia Appendix 2 [file jmir_v27i1e63975_app2.docx]

**Table S1**.

| **Prediabetes phenotype** | **Diagnostic criteria** | **FPG^a^** | **2-h PG^b^** | **HbA1c** |
| --- | --- | --- | --- | --- |
| IFG^c^ | ADA^d^(2003)[73]  ADA(2010)[74] | 100-125mg/dL  (5.6-6.9mmol/L) | - | - |
| IFG | WHO^e^(1999)[75] | 110-125mg/dL  (6.1-6.9mmol/L) | (if measured)  ＜140mg/dL  (7.8mmol/L) | - |
| IGT^f^ | WHO(1999)[75]  ADA(2003)[73]  ADA(2010)[74] | 140-199mg/dL  (7.8-11.0mmol/L) | - | - |
| IGT | WHO(1980)[76] | ＜140mg/dL  (7.8 mmol/L) | 140-199mg/dL  (7.8~11.0mmol/L) |  |
| IGT | Adapted WHO(1980)[76] | 95-125mg/dL  (5.3-6.9mmol/L) | 140-199mg/dL  (7.8~11.0mmol/L) |  |
| Elevated HbA1c^g^ | ADA(2010)[74] | - | - | 5.7-6.4%  (39-46 mmol/mol) |
| Elevated HbA1c | IEC^h^(2009)[77] | - | - | 6.0–6.4 %  (42-46 mmol/mol) |
| IH^i^ | ADA(1997)[78] | 95-125mg/dL  5.3~6.9 | 140-199mg/dL  (7.8~11.0mmol/L) |  |

^a^FPG: Fasting Plasma Glucose; ^b^2h-PG: 2-h Plasma Glucose after oral glucose tolerance test; ^c^IFG:Impaired Fasting Glucose; ^d^ADA: American Diabetes Association; ^e^WHO: World Health Organization; ^f^IGT: Impaired Glucose Tolerance; ^g^HbA1c: Hemoglobin A1c; ^h^IEC: The International Expert Committee; ^i^IH: Intermediate Hyperglycemia

73. Diagnosis and classification of diabetes mellitus. Diabetes Care. 2004 Jan;27 Suppl 1:S5-s10. PMID: 14693921. doi: 10.2337/diacare.27.2007.s5.

74. Diagnosis and classification of diabetes mellitus. Diabetes Care. 2010 Jan;33 Suppl 1(Suppl 1):S62-9. PMID: 20042775. doi: 10.2337/dc10-S062.

75. World Health O. Definition, diagnosis and classification of diabetes mellitus and its complications : report of a WHO consultation. Part 1, Diagnosis and classification of diabetes mellitus. Geneva: World Health Organization; 1999.

76. WHO Expert Committee on Diabetes Mellitus: second report. World Health Organ Tech Rep Ser. 1980;646:1-80. PMID: 6771926.

77. International Expert Committee report on the role of the A1C assay in the diagnosis of diabetes. Diabetes Care. 2009 Jul;32(7):1327-34. PMID: 19502545. doi: 10.2337/dc09-9033.

78. Diagnosis TECot, Mellitus CoD. Report of the Expert Committee on the Diagnosis and Classification of Diabetes Mellitus. Diabetes Care. 1997;20(7):1183-97. doi: 10.2337/diacare.20.7.1183.
