# Supplementary material for: Effectiveness of Different Intervention Modes in Lifestyle Intervention for the Prevention of Type 2 Diabetes and the Reversion to Normoglycemia in Adults With Prediabetes: Systematic Review and Meta-Analysis of Randomized Controlled Trials
Source: J Med Internet Res. 2025 Jan 29;27:e63975. doi: 10.2196/63975 (PMC11822313; doi:10.2196/63975)
Supplement: Multimedia Appendix 3 [file jmir_v27i1e63975_app3.docx]

**Multimedia Appendix 3 Characteristics of the included studies**

Table S3-1 Characteristics of the studies at baseline investigating the effectiveness of lifestyle intervention on prediabetes

Table S3-2 Intervention characteristics of the studies investigating the effectiveness of lifestyle intervention on prediabetes

Table S3-3 Intervention characteristics of the studies investigating the effects of studies including digital health intervention components

Table S3-1 Characteristics of the studies at baseline investigating the effectiveness of lifestyle intervention on prediabetes

| First author, publication year | Prediabetes phenotype | Diagnostic criteria | Location | Age, years  mean(SD) | Male,% | BMI^a^ , kg/m²  mean(SD) | FPG^b^, mmol/L  mean(SD) | Sample size |
| --- | --- | --- | --- | --- | --- | --- | --- | --- |
| Aekplakorn, 2019[1] | IGT^c^ and/or IFG^d^ | WHO^e^(1999) | Thailand | 50.9(6.39) | 20.28 | 27.0(4.60) | 5.4(0.69) | 1903 |
| Bhopal, 2014[2] | IGT and/or IFG | WHO(1999) | UK | 52.5(10.22) | 45.61 | 30.6(4.79) | 5.8(0.60) | 171 |
| Chen, 2021[3] | IGT or IFG or elevated HbA1c | ADA^k^(2016) | China | 60.5(5.81) | 35.08 | 24.8(2.80) | NA^f^ | 248 |
| Da Qing, 1997[4] | IGT and/or IFG | WHO(1985) | China | 45.0(9.10) | 53.40 | 25.8(3.80) | 5.6(0.81) | 530 |
| Davies, 2016[5] | IGT or IFG | WHO(1999) | UK | 63.9(7.74) | 63.64 | 32.5(5.50) | 5.7(0.70) | 880 |
| DPP, 2002[6] | IGT and/or IFG | Adapt WHO(1980)* | US | 50.5(10.86) | 31.47 | 34.1(6.75) | 5.9(0.46) | 2161 |
| DPS, 2001[7] | IGT and/or IFG | WHO(1985) | Finland | 55.0(7.00) | 32.95 | 31.2(4.55) | 6.1(0.75) | 522 |
| Fottrell, 2019[8] | IGT and/or IFG | WHO(1999) | Bangladesh | NA | NA | NA | NA | 2470 |
| Gagnon, 2011[9] | IFG or IGT | WHO(1999) | Canada | 56.8(11.20) | 54.17 | 35.1(5.50) | 5.9(0.60) | 48 |
| Hellgren, 2016[10] | IGT and/or IFG | WHO(1999) | Sweden | 63.0(9.00) | 41.67 | 30.0(4.40) | NA | 96 |
| Hu, 2017[11] | IGT and/or IFG | WHO(1999) | China | 69.4(6.55) | 41.47 | 23.7(3.46) | 6.1(0.55) | 434 |
| Iqbal Hydrie, 2012[12] | IGT and/or IFG | WHO(1999) | Pakistan | 43.6(10.49) | NA | 26.5(5.22) | NA | 222 |
| Katula, 2013[13] | IFG and/or IGT | Adapt WHO(1980) | US | 57.9(9.50) | 42.52 | 32.7(4.00) | 5.9(0.63) | 301 |
| Kosaka, 2005[14] | IGT and/or IFG | WHO(1985) | Japan | NA | 100.00 | 23.8(2.15) | NA | 458 |
| Liu, 2022[15] | IGT and/or IFG plus elevated HbA1c | WHO(1999) plus elevated HbA1c | China | 52.7(8.57) | NA | 25.7(3.51) | 5.9(0.43) | 2865 |
| Luo, 2022[16] | IGT and/or IFG | WHO(1999) | China | NA | 40.82 | NA | NA | 970 |
| Nanditha, 2020[17] | Only elevated HbA1c | ADA(2016) | UK&India | 52.05(10.25) | 63.97 | 28.8(4.75) | 5.5(0.70) | 2062 |
| Penn, 2009[18] | IGT and/or IFG | WHO(1999) | UK | NA | 40.20 | 33.8(5.05) | 5.8(0.55) | 102 |
| Ramachandran, 2006[19] | IGT and/or IFG | WHO(1999) | India | 45.7(5.71) | 77.32 | 26.0(3.51) | 5.5(0.75) | 269 |
| Roumen, 2008[20] | IGT and/or IFG | ADA(2003) | Dutch | 56.9(7.72) | 51.02 | 29.8(3.80) | 6.0(0.77) | 147 |
| Saito, 2011[21] | IFG or/and IGT | ADA(2016) | Japan | NA | 22.31 | 27.0(2.60) | 6.0(8.01) | 641 |
| Sakane, 2011[22] | IGT and/or IFG | WHO(1999) | Japan | 51.0(6.50) | 49.34 | 24.7(3.40) | 6.0(0.51) | 304 |
| Sakane, 2015[23] | IFG and/or IGT | ADA(2003) | Japan | 48.9(7.64) | 83.35 | 24.4(3.15) | NA | 2607 |
| Sampson, 2021[24] | IFG and/or elevated HbA1c | ADA(2016) | UK | 66.4(9.24) | 62.74 | 31.0(5.50) | 6.2(0.40) | 1028 |
| Shahbazi, 2017[25] | IGT or IFG | ADA(2003) | Iran | 43.6(9.74) | 34.78 | NA | NA | 322 |
| Staite, 2020[26] | Only elevated HbA1c | ADA(2016) | UK | 52.3 (7.94) | 47.00 | NA | NA | 200 |
| Thankappan, 2018[27] | IGT and/or IFG | ADA(2003) | India | NA | NA | NA | NA | 695 |
| Wani, 2020[28] | IFG and/or IGT | ADA(2003) | Saudi Arabia | 43.4(10.17) | 30.67 | 32.3(6.02) | 6.1(0.51) | 300 |
| Wong, 2013[29] | IFG or IGT | ADA(2016) | Chinese HK | 54.6(6.29) | 93.27 | 25.9(2.95) | 5.9(0.45) | 104 |
| Yates, 2009[30] | IGT and/or IFG | WHO(1999) | UK | 65(8.00) | 65.52 | 29.2(4.70) | 5.6(0.60) | 87 |
| Ramachandran, 2013[31] | IGT or IFG | WHO(1999) | India | 46.0(4.70) | 100.00 | 25.8(3.15) | 5.7(0.54) | 537 |

^a^BMI:body mass index; ^b^FPG:fasting plasma glucose; ^c^IGT:impaired glucose tolerance; ^d^IFG:impaired fasting glucose; ^e^WHO:World Health Organization; ^f^NA:Unknown; ^g^ADA:American Diabetes Association

Table S3-2 Intervention characteristics of the studies investigating the effectiveness of lifestyle intervention on prediabetes

| First Author, Publication year | Treatment | Control | Settings | Delivery format | Intervention mode | Intervention duration, years | Follow-up duration, years^a^ |
| --- | --- | --- | --- | --- | --- | --- | --- |
| Aekplakorn, 2019[1] | diet and physical activity | receive a one-time education program | community | group sessions | face-to-face interventions | 2 | 2 |
| Bhopal, 2014[2] | diet and physical activity | receive standard health care advice | family | consultation with a dietitian | face-to-face interventions | 3 | 3 |
| Chen, 2021[3] | only physical activity | receive no structured exercise intervention or counseling | clinic | group sessions | face-to-face interventions | 2 | 2 |
| Da Qing, 1997[4] | diet/physical activity/diet and physical activity | receive standard health care advice | clinic | group sessions | face-to-face interventions | 6 | 6 |
| DPP, 2002[6] | diet and physical activity | standard lifestyle recommendations plus placebo twice daily | clinic | group sessions | face-to-face interventions | 2.8 | 2.8 |
| DPS, 2001[7] | diet and physical activity | receive standard health care advice | Not specific | group sessions | face-to-face interventions | 4 | 3.2 |
| Fottrell, 2019[8] | diet and physical activity | receive standard health care advice | community | group sessions | face-to-face interventions | 1.6 | 2 |
| Gagnon, 2011[9] | diet and physical activity | enhanced usual care | clinic | group sessions | face-to-face interventions | 1 | 1 |
| Hu, 2017[11] | diet and physical activity | receive standard health care advice | community | group sessions | face-to-face interventions | 1 | 1 |
| Iqbal Hydrie, 2012[12] | only physical activity | receive standard health care advice | clinic | group sessions | face-to-face interventions | 1.5 | 1.5 |
| Katula, 2013[13] | diet and physical activity | receive two visits with a registered dietitian and a monthly  newsletter | community | group sessions | face-to-face interventions | 2 | 2 |
| Kosaka, 2005[14] | diet and physical activity | receive standard health care advice | clinic | questions and instructions | face-to-face interventions | 4 | 4 |
| Liu, 2022[15] | diet and physical activity | receive standard health care advice | clinic | group sessions | face-to-face interventions | 1 | 1 |
| Luo, 2022[16] | diet and physical activity | receive standard health care advice | clinic | group sessions | face-to-face interventions | 3 | 3 |
| Penn, 2009[18] | diet and physical activity | receive standard health care advice | clinic | individual advice from a dietician and physiotherapist group sessions regular quarterly newsletter. | face-to-face interventions | 5 | 5 |
| Roumen, 2008[20] | diet and physical activity | receive standard health care advice | Not specific | counselling session | face-to-face interventions | 3 | 3 |
| Saito, 2011[21] | diet and physical activity | receive similar individual instructions 4 times at baseline and 12, 24, and 36 months | clinic | individual instructions and follow-up support | face-to-face interventions | 3 | 3 |
| Sakane, 2011[22] | diet and physical activity | received only one group session at the baseline | clinic | both group and individual sessions | face-to-face interventions | 3 | 3 |
| Shahbazi, 2017[25] | only diet | receive standard health care advice | clinic | individualized counseling | face-to-face interventions | 2 | 2 |
| Thankappan, 2018[27] | diet and physical activity | receive standard health care advice | community | group sessions | face-to-face interventions | 2 | 2 |
| Davies, 2016[5] | diet and physical activity | receive standard health care advice | community | re-fresh sessions  telephone contacts  pedometer | blended support interventions | 3 | 3 |
| Fottrell, 2019[8] | diet and physical activity | receive standard health care advice | digital | voice messages | digital support interventions | 1.2 | 2 |
| Hellgren, 2016[10] | only physical activity | receive standard health care advice | community and digital | group sessions telephone contacts(begin at third year) | blended support interventions | 3 | 3 |
| Nanditha, 2020[17] | diet and physical activity | receive standard health care advice | digital | received regular SMS^b^ messages, typically to provide additional education and motivation. | digital support interventions | 2 | 2 |
| Ramachandran, 2006[19] | diet and physical activity | receive standard health care advice | community | telephonic contacts personal sessions | blended support interventions | 3 | 3 |
| Sakane, 2015[23] | diet and physical activity | receive standard health care advice | digital | Newsletters  Advice mail telephone contacts | digital support interventions | 1 | 4.2 |
| Sampson, 2021[24] | diet and physical activity | receive standard health care advice | clinic and digital | group sessions telephone contacts | blended support interventions | 3.8 | 3.8 |
| Staite, 2020[26] | diet and physical activity | receive the wearable technology and access to the web-based DDP but not the SMS texts | digital | wearable Technology web-based Education motivation SMS tests | digital support interventions | 1 | 1 |
| Wani, 2020[28] | diet and physical activity | receive standard health care advice | clinic | Education workshops  individual consultation and SMS/telephone contacts and pedometers | blended support interventions | 1 | 1 |
| Wong, 2013[29] | diet and physical activity | receive standard health care advice | digital | SMS | digital support interventions | 2 | 2 |
| Yates, 2009[30] | only physical activity | receive standard health care advice | clinic | Education workshops  provided with a pedometer | blended support interventions | 1 | 1 |
| Ramachandran, 2013[31] | diet and physical activity | receive standard health care advice | community | SMS | digital support interventions | 2 | 2 |

^a^Time to assess diabetes incidence/reversion to normoglycemia; ^b^SMS:short message service

Table S3-3 Intervention characteristics of the studies investigating the effects of studies including digital health intervention components

| First Author, Publication year | Theory | Adherence | Digital health category | Interventions |
| --- | --- | --- | --- | --- |
| Davies, 2016[5] | Self-regulatory strategies | Not specific | - 1. Targeted client communication   1.4 Personal health track | 1.1.2 Transmit targeted health information to client based on health status or demographics  1.4.2 Self monitoring of health or diagnostic data by client |
| Fottrell, 2019[8] | Formative research and behaviour change theories | Not specific | 1.1 Targeted client communication | 1.1.2 Transmit targeted health information to client based on health status or demographics |
| Hellgren, 2016[10] | Not specific | Not specific | 1.1 Targeted client communication | 1.1.2 Transmit targeted health information to client based on health status or demographics |
| Nanditha, 2020[17] | The transtheoretical model of behavioural change | Not specific | 1.1 Targeted client communication | 1.1.2 Transmit targeted health information to client based on health status or demographics |
| Ramachandran, 2006[19] | Not specific | 41.7% to 58.8% | 1.1 Targeted client communication | 1.1.2 Transmit targeted health information to client based on health status or demographics |
| Sakane, 2015[23] | Self-monitoring of personal action plan  Self-assessment  Feedback  Personal Action Plan  Problem-solving  Self-efficacy  Motivation for lifestyle modifications  Knowledge | The rates of good adherence were 91.4%, 82.7% and 81.1%, for each centre, respectively | 1.1 Targeted client communication | 1.1.2 Transmit targeted health information to client based on health status or demographics |
| Sampson, 2021[24] | Self Determination Theory, Social Cognitive Theory, Control Theory and the Health Action Process Approach | Not specific | 1.1 Targeted client communication | 1.1.2 Transmit targeted health information to client based on health status or demographics |
| Staite, 2020[26] | MI techniques  The theory of planned behavior  Intention formation | Not specific | 1.1 Targeted client communication  1.4 Personal health track  2.4 Telemedicine | 1.1.2 Transmit targeted health information to client based on health status or demographics  1.4.1 Access by client to own medical records  1.4.2 Self monitoring of health or diagnostic data by client  1.4.3 Active data capture/Documentation by client  2.4.2 Remote monitoring of client health or diagnostic data by provider  2.4.3 Transmission of medical data |
| Wani, 2020[28] | Not specific | Not specific | 1.1 Targeted client communication  1.4 Personal health track | 1.1.2 Transmit targeted health information to client based on health status or demographics  1.4.2 Self monitoring of health or diagnostic data by client |
| Wong, 2013[29] | The theory of planned behavior Social cognitive theory | Not specific | 1.1 Targeted client communication | 1.1.2 Transmit targeted health information to client based on health status or demographics |
| Yates, 2009[30] | Social cognitive theory  Leventhal's common sense model | Not specific | 1.4 Personal health track | 1.4.2 Self monitoring of health or diagnostic data by client |
| Ramachandran, 2013[31] | The transtheoretical model of behavioural change | Not specific | 1.1 Targeted client communication | 1.1.2 Transmit targeted health information to client based on health status or demographics |

1. Aekplakorn W, Tantayotai V, Numsangkul S, Tatsato N, Luckanajantachote P, Himathongkam T. Evaluation of a Community-Based Diabetes Prevention Program in Thailand: A Cluster Randomized Controlled Trial. J Prim Care Community Health. 2019 Jan-Dec;10:2150132719847374. PMID: 31079517. doi: 10.1177/2150132719847374.

2. Bhopal RS, Douglas A, Wallia S, Forbes JF, Lean ME, Gill JM, et al. Effect of a lifestyle intervention on weight change in south Asian individuals in the UK at high risk of type 2 diabetes: a family-cluster randomised controlled trial. Lancet Diabetes Endocrinol. 2014 Mar;2(3):218-27. PMID: 24622752. doi: 10.1016/s2213-8587(13)70204-3.

3. Chen X, Zhao S, Hsue C, Dai X, Liu L, Miller JD, et al. Effects of aerobic training and resistance training in reducing cardiovascular disease risk for patients with prediabetes: A multi-center randomized controlled trial. Prim Care Diabetes. 2021 Dec;15(6):1063-70. PMID: 34649825. doi: 10.1016/j.pcd.2021.08.013.

4. Pan XR, Li GW, Hu YH, Wang JX, Yang WY, An ZX, et al. Effects of diet and exercise in preventing NIDDM in people with impaired glucose tolerance. The Da Qing IGT and Diabetes Study. Diabetes Care. 1997 Apr;20(4):537-44. PMID: 9096977. doi: 10.2337/diacare.20.4.537.

5. Davies MJ, Gray LJ, Troughton J, Gray A, Tuomilehto J, Farooqi A, et al. A community based primary prevention programme for type 2 diabetes integrating identification and lifestyle intervention for prevention: the Let's Prevent Diabetes cluster randomised controlled trial. Prev Med. 2016 Mar;84:48-56. PMID: 26740346. doi: 10.1016/j.ypmed.2015.12.012.

6. Knowler WC, Barrett-Connor E, Fowler SE, Hamman RF, Lachin JM, Walker EA, et al. Reduction in the incidence of type 2 diabetes with lifestyle intervention or metformin. N Engl J Med. 2002 Feb 7;346(6):393-403. PMID: 11832527. doi: 10.1056/NEJMoa012512.

7. Tuomilehto J, Lindström J, Eriksson JG, Valle TT, Hämäläinen H, Ilanne-Parikka P, et al. Prevention of type 2 diabetes mellitus by changes in lifestyle among subjects with impaired glucose tolerance. N Engl J Med. 2001 May 3;344(18):1343-50. PMID: 11333990. doi: 10.1056/nejm200105033441801.

8. Fottrell E, Ahmed N, Morrison J, Kuddus A, Shaha SK, King C, et al. Community groups or mobile phone messaging to prevent and control type 2 diabetes and intermediate hyperglycaemia in Bangladesh (DMagic): a cluster-randomised controlled trial. The lancet Diabetes & endocrinology. 2019;7(3):200. doi: https://doi.org/10.1016/S2213-8587(19)30001-4.

9. Gagnon C, Brown C, Couture C, Kamga-Ngande CN, Hivert MF, Baillargeon JP, et al. A cost-effective moderate-intensity interdisciplinary weight-management programme for individuals with prediabetes. Diabetes Metab. 2011 Nov;37(5):410-8. PMID: 21489843. doi: 10.1016/j.diabet.2011.01.003.

10. Hellgren MI, Jansson PA, Wedel H, Lindblad U. A lifestyle intervention in primary care prevents deterioration of insulin resistance in patients with impaired glucose tolerance: A randomised controlled trial. Scand J Public Health. 2016 Nov;44(7):718-25. PMID: 27550085. doi: 10.1177/1403494816663539.

11. Hu Z, Qin L, Xu H. One-Year Results of a Synthetic Intervention Model for the Primary Prevention of T2D among Elderly Individuals with Prediabetes in Rural China. Int J Environ Res Public Health. 2017 Apr 14;14(4). PMID: 28420105. doi: 10.3390/ijerph14040417.

12. Iqbal Hydrie MZ, Basit A, Shera AS, Hussain A. Effect of Intervention in Subjects with High Risk of Diabetes Mellitus in Pakistan. Journal of Nutrition and Metabolism. 2012;2012:1-7. doi: 10.1155/2012/867604.

13. Katula JA, Vitolins MZ, Morgan TM, Lawlor MS, Blackwell CS, Isom SP, et al. The Healthy Living Partnerships to Prevent Diabetes study: 2-year outcomes of a randomized controlled trial. Am J Prev Med. 2013 Apr;44(4 Suppl 4):S324-32. PMID: 23498294. doi: 10.1016/j.amepre.2012.12.015.

14. Kosaka K, Noda M, Kuzuya T. Prevention of type 2 diabetes by lifestyle intervention: a Japanese trial in IGT males. Diabetes Res Clin Pract. 2005 Feb;67(2):152-62. PMID: 15649575. doi: 10.1016/j.diabres.2004.06.010.

15. Liu Y, Guo H, Wang Q, Chen J, Xuan Y, Xu J, et al. Short-term effects of lifestyle intervention in the reversion to normoglycemia in people with prediabetes. Prim Care Diabetes. 2022 Feb;16(1):168-72. PMID: 34930688. doi: 10.1016/j.pcd.2021.12.009.

16. Luo Y, Wang H, Zhou X, Chang C, Chen W, Guo X, et al. A Randomized Controlled Clinical Trial of Lifestyle Intervention and Pioglitazone for Normalization of Glucose Status in Chinese with Prediabetes. J Diabetes Res. 2022;2022:2971382. PMID: 35036447. doi: 10.1155/2022/2971382.

17. Nanditha A, Thomson H, Susairaj P, Srivanichakorn W, Oliver N, Godsland IF, et al. A pragmatic and scalable strategy using mobile technology to promote sustained lifestyle changes to prevent type 2 diabetes in India and the UK: a randomised controlled trial. Diabetologia. 2020 Mar;63(3):486-96. PMID: 31919539. doi: 10.1007/s00125-019-05061-y.

18. Penn L, White M, Oldroyd J, Walker M, Alberti KG, Mathers JC. Prevention of type 2 diabetes in adults with impaired glucose tolerance: the European Diabetes Prevention RCT in Newcastle upon Tyne, UK. BMC Public Health. 2009 Sep 16;9:342. PMID: 19758428. doi: 10.1186/1471-2458-9-342.

19. Ramachandran A, Snehalatha C, Mary S, Mukesh B, Bhaskar AD, Vijay V. The Indian Diabetes Prevention Programme shows that lifestyle modification and metformin prevent type 2 diabetes in Asian Indian subjects with impaired glucose tolerance (IDPP-1). Diabetologia. 2006 Feb;49(2):289-97. PMID: 16391903. doi: 10.1007/s00125-005-0097-z.

20. Roumen C, Corpeleijn E, Feskens EJ, Mensink M, Saris WH, Blaak EE. Impact of 3-year lifestyle intervention on postprandial glucose metabolism: the SLIM study. Diabet Med. 2008 May;25(5):597-605. PMID: 18445174. doi: 10.1111/j.1464-5491.2008.02417.x.

21. Saito T, Watanabe M, Nishida J, Izumi T, Omura M, Takagi T, et al. Lifestyle modification and prevention of type 2 diabetes in overweight Japanese with impaired fasting glucose levels: a randomized controlled trial. Arch Intern Med. 2011 Aug 8;171(15):1352-60. PMID: 21824948. doi: 10.1001/archinternmed.2011.275.

22. Sakane N, Kotani K, Okazaki K, Sato J, Suzuki S, Morita S, et al. Recruitment and baseline characteristics of J-DOIT1 (Japan diabetes outcome trial 1). Diabetes Technology and Therapeutics. 2011;13(2):267-8. doi: https://dx.doi.org/10.1089/dia.2010.1219.

23. Sakane N, Kotani K, Takahashi K, Sano Y, Tsuzaki K, Okazaki K, et al. Effects of telephone-delivered lifestyle support on the development of diabetes in participants at high risk of type 2 diabetes: J-DOIT1, a pragmatic cluster randomised trial. BMJ Open. 2015 Aug 19;5(8):e007316. PMID: 26289448. doi: 10.1136/bmjopen-2014-007316.

24. Sampson M, Clark A, Bachmann M, Garner N, Irvine L, Howe A, et al. Lifestyle Intervention With or Without Lay Volunteers to Prevent Type 2 Diabetes in People With Impaired Fasting Glucose and/or Nondiabetic Hyperglycemia: A Randomized Clinical Trial. JAMA Intern Med. 2021 Feb 1;181(2):168-78. PMID: 33136119. doi: 10.1001/jamainternmed.2020.5938.

25. Shahbazi S, Vahdat Shariatpanahi Z. Prevention of type 2 diabetes mellitus by changes in diet among subjects with abnormal glucose metabolism: a randomized clinical trial. International Journal of Diabetes in Developing Countries. 2018 2018/01/01;38(1):69-74. doi: 10.1007/s13410-017-0548-3.

26. Staite E, Bayley A, Al-Ozairi E, Stewart K, Hopkins D, Rundle J, et al. A Wearable Technology Delivering a Web-Based Diabetes Prevention Program to People at High Risk of Type 2 Diabetes: Randomized Controlled Trial. JMIR Mhealth Uhealth. 2020 Jul 15;8(7):e15448. PMID: 32459651. doi: 10.2196/15448.

27. Thankappan KR, Sathish T, Tapp RJ, Shaw JE, Lotfaliany M, Wolfe R, et al. A peer-support lifestyle intervention for preventing type 2 diabetes in India: A cluster-randomized controlled trial of the Kerala Diabetes Prevention Program. PLoS Med. 2018 Jun;15(6):e1002575. PMID: 29874236. doi: 10.1371/journal.pmed.1002575.

28. Wani K, Alfawaz H, Alnaami AM, Sabico S, Khattak MNK, Al-Attas O, et al. Effects of A 12-Month Intensive Lifestyle Monitoring Program in Predominantly Overweight/Obese Arab Adults with Prediabetes. Nutrients. 2020 Feb 12;12(2). PMID: 32059477. doi: 10.3390/nu12020464.

29. Wong CK, Fung CS, Siu SC, Lo YY, Wong KW, Fong DY, et al. A short message service (SMS) intervention to prevent diabetes in Chinese professional drivers with pre-diabetes: a pilot single-blinded randomized controlled trial. Diabetes Res Clin Pract. 2013 Dec;102(3):158-66. PMID: 24466598. doi: 10.1016/j.diabres.2013.10.002.

30. Yates T, Davies M, Gorely T, Bull F, Khunti K. Effectiveness of a pragmatic education program designed to promote walking activity in individuals with impaired glucose tolerance: a randomized controlled trial. Diabetes Care. 2009 Aug;32(8):1404-10. PMID: 19602539. doi: 10.2337/dc09-0130.

31. Ramachandran A, Snehalatha C, Ram J, Selvam S, Simon M, Nanditha A, et al. Effectiveness of mobile phone messaging in prevention of type 2 diabetes by lifestyle modification in men in India: a prospective, parallel-group, randomised controlled trial. Lancet Diabetes Endocrinol. 2013 Nov;1(3):191-8. PMID: 24622367. doi: 10.1016/s2213-8587(13)70067-6.
