# Supplementary material for: Effectiveness of Different Intervention Modes in Lifestyle Intervention for the Prevention of Type 2 Diabetes and the Reversion to Normoglycemia in Adults With Prediabetes: Systematic Review and Meta-Analysis of Randomized Controlled Trials
Source: J Med Internet Res. 2025 Jan 29;27:e63975. doi: 10.2196/63975 (PMC11822313; doi:10.2196/63975)
Supplement: Multimedia Appendix 4 [file jmir_v27i1e63975_app4.docx]

Assessment of Secondary Outcomes, Evaluation of Publication Bias, and Sensitivity Analysis

**Table S1. Effect of secondary outcome by subgroup**

| Group | SMD(95%CI) | *I*^2^(%) | *P* value |
| --- | --- | --- | --- |
| Weight loss |  |  |  |
| Face-to-face | -0.25(-0.35 to -0.15) | 48 | ＜.001 |
| Digital | -0.07(-0.17 to 0.03) | 48 | .15 |
|  |  |  |  |
| WC |  |  |  |
| Face-to-face | -0.31(-0.54,-0.08) | 80 | .009 |
| Digital | 0.02(-0.06,0.09) | 0 | .69 |
|  |  |  |  |
| BMI |  |  |  |
| Face-to-face | -0.33(-0.50 to -0.15) | 84 | ＜.001 |
| Digital | 0.01(-0.06 to 0.09) | 0 | .72 |
|  |  |  |  |
| SBP |  |  |  |
| Face-to-face | -0.10(-0.18 to -0.01) | 36 | .02 |
| Digital | 0.01(-0.06 to 0.08) | 0 | .78 |
|  |  |  |  |
| DBP |  |  |  |
| Face-to-face | -0.10(-0.20 to -0.01) | 48 | .04 |
| Digital | 0.03(-0.26 to 0.32) | 86 | .84 |
|  |  |  |  |
| TC |  |  |  |
| Face-to-face | 0.05(-0.28 to 0.38) | 86 | .77 |
| Digital | 0.01(-0.06 to 0.08) | 0 | .78 |
|  |  |  |  |
| TG |  |  |  |
| Face-to-face | -0.28(-0.52 to -0.04) | 93 | .02 |
| Digital | 0.06(-0.26 to 0.38) | 24 | .69 |
|  |  |  |  |
| HDL-C |  |  |  |
| Face-to-face | 0.13(-0.02 to 0.28) | 70 | .09 |
| Digital | 0.14(-0.18 to 0.45) | 89 | .39 |
|  |  |  |  |
| LDL-C |  |  |  |
| Face-to-face | -0.13(-0.41 to 0.14) | 81 | .34 |
| Digital | -0.10(-0.19 to -0.02) | 0 | .01 |
|  |  |  |  |
| FPG |  |  |  |
| Face-to-face | -0.32(-0.39 to -0.26) | 50 | ＜.001 |
| Digital | 0.00(-0.15 to 0.16） | 50 | .50 |
|  |  |  |  |
| 2hPG |  |  |  |
| Face-to-face | -0.30(-0.46 to -0.14) | 87 | ＜.001 |
| Digital | -0.21(-0.60 to 0.18) | 67 | .88 |
|  |  |  |  |
| HbA1c |  |  |  |
| Face-to-face | -0.30(-0.57 to -0.02) | 74 | .03 |
| Digital | 0.05(-0.13 to 0.23) | 85 | .58 |

Risk bias assessment and funnel plot of publication bias


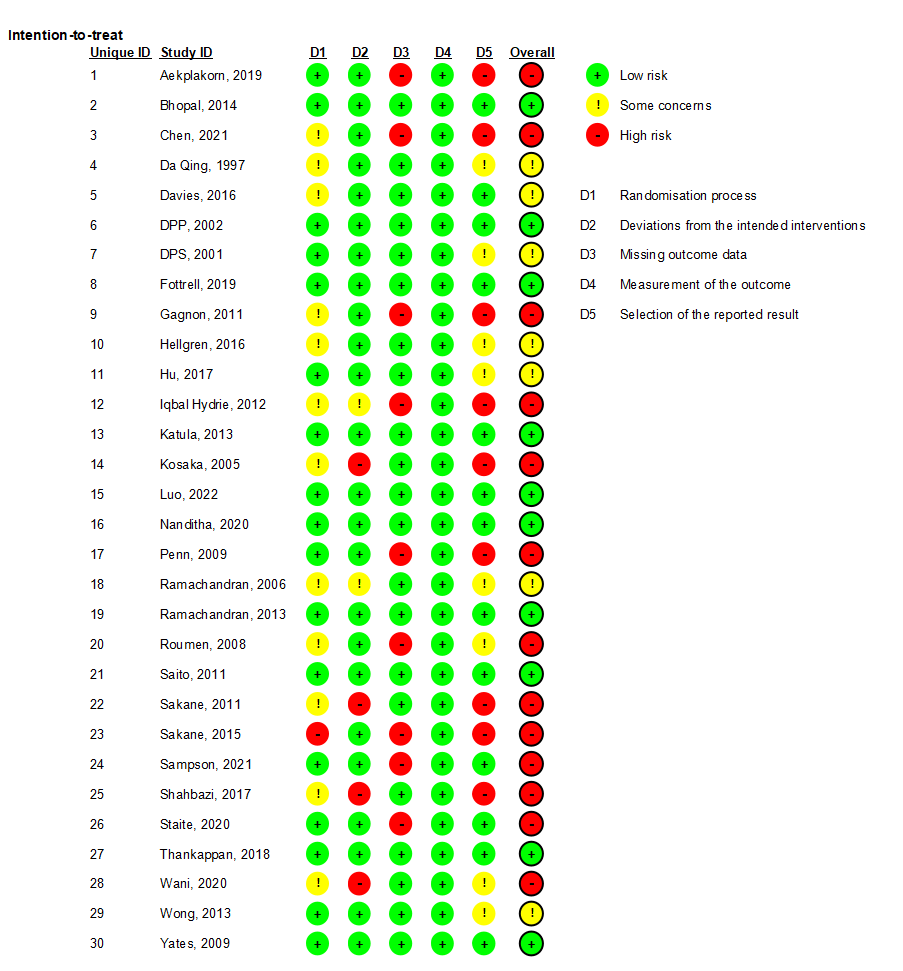


**Figure S1.** ROB-2 bias assessment of included studies of T2DM incidence. Red:high risk; Yellow:Unclear risk; Green:Low risk


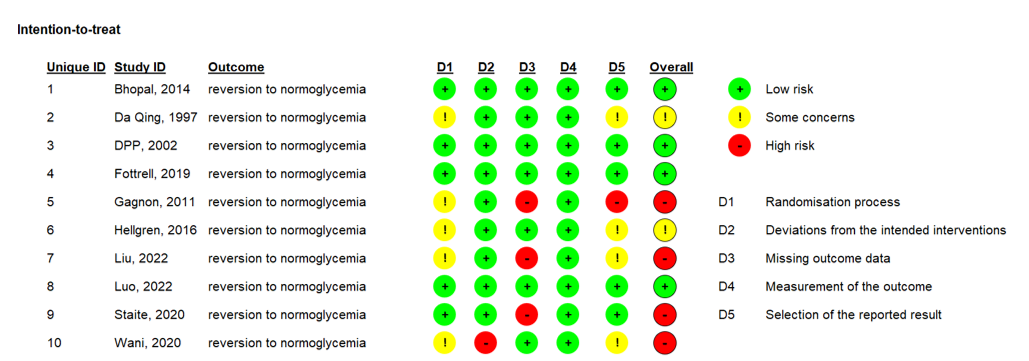


**Figure S2.** ROB-2 bias assessment of included studies of reversion to normoglycemia.


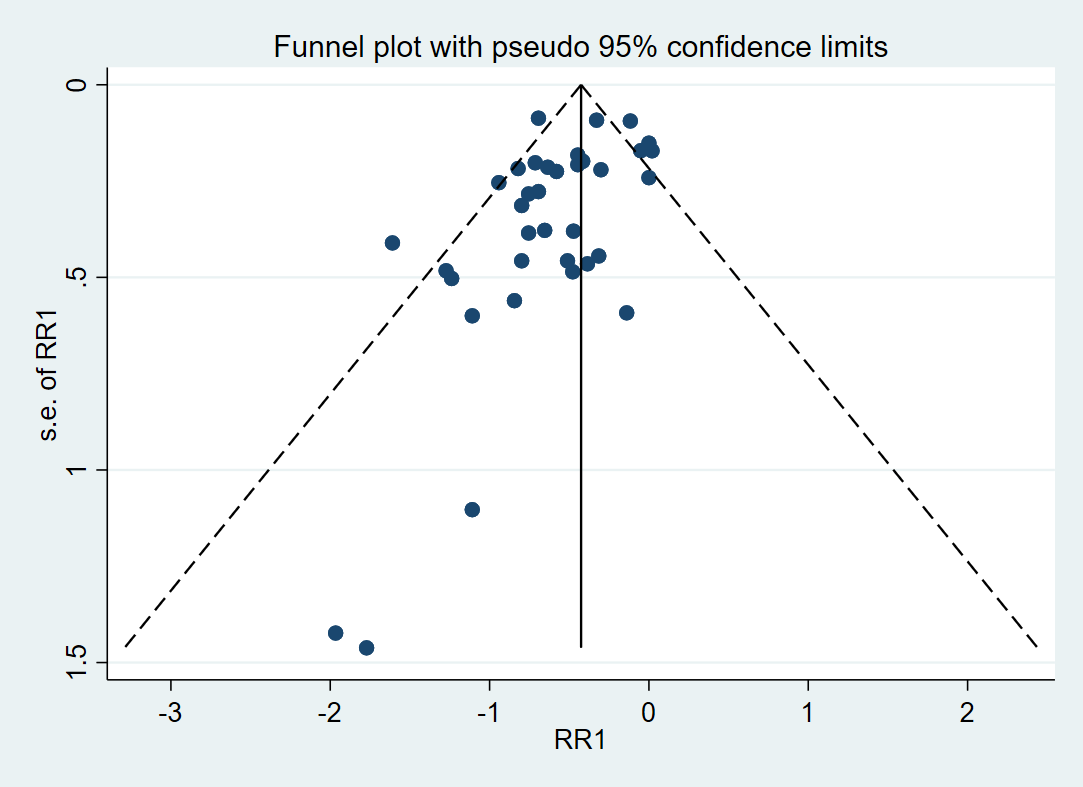


**Figure S3.** Funnel plot of studies of lifestyle intervention for diabetes prevention among prediabetes.


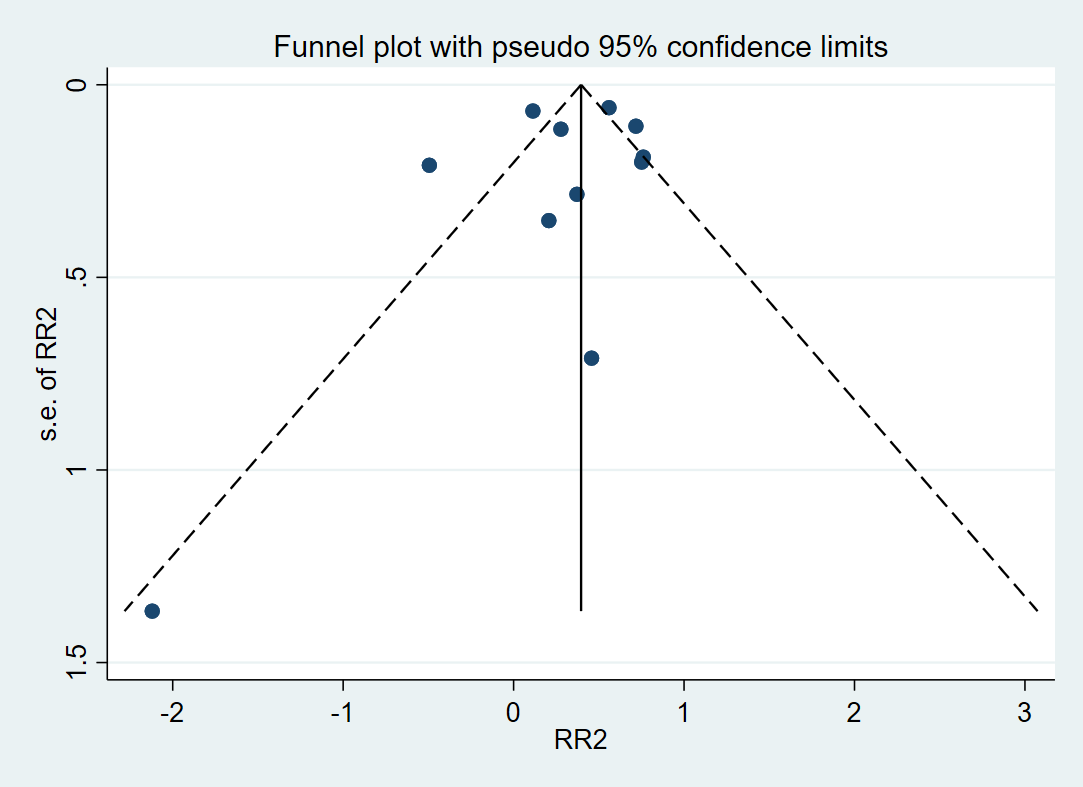


**Figure S4.** Funnel plot of studies of lifestyle intervention for hyperglycemia reversion among prediabetes.

Sensitive analysis


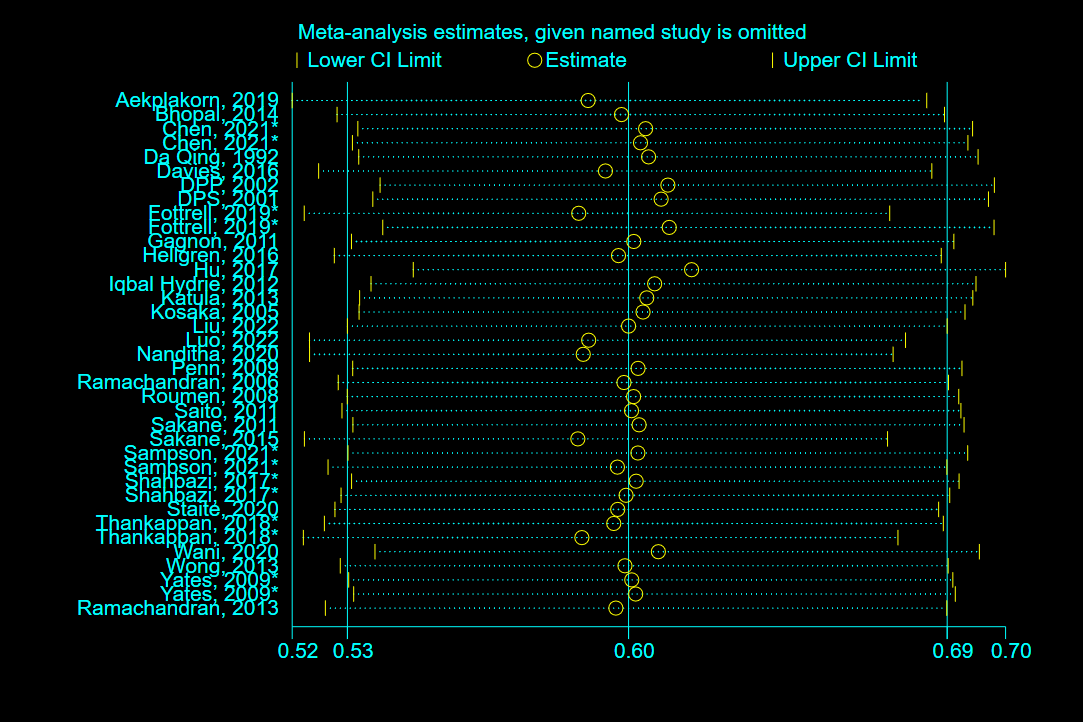


**Figure S5.** Sensitive analysis of studies of lifestyle intervention for diabetes prevention among prediabetes.


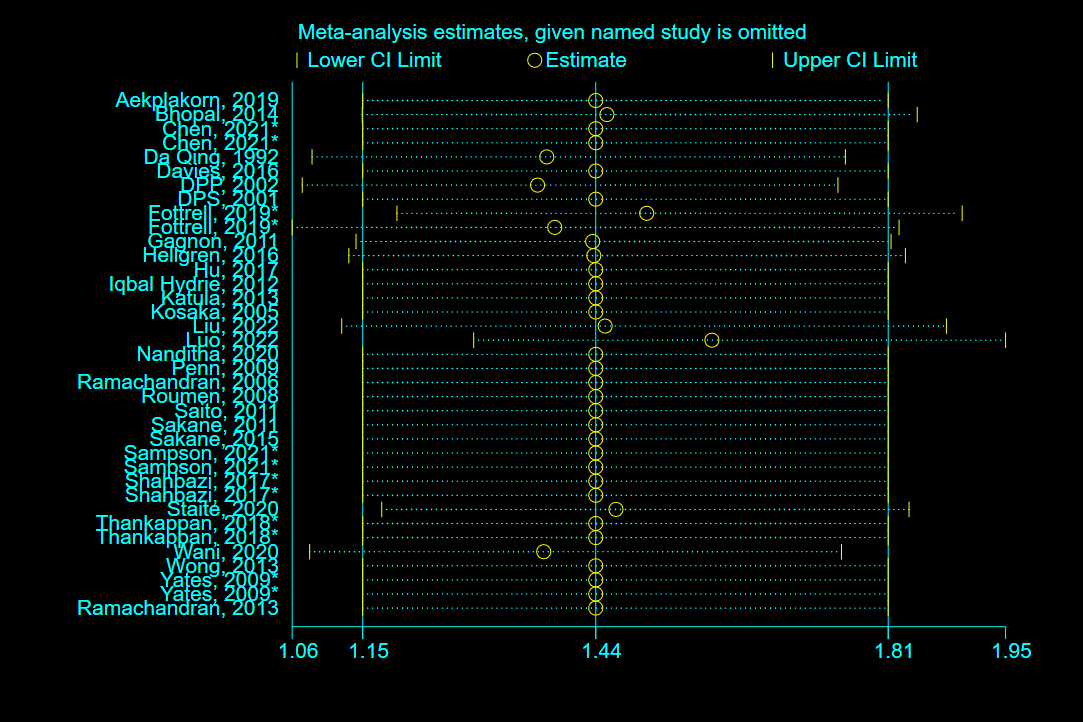


**Figure S6.** Sensitive analysis of studies of lifestyle intervention for diabetes prevention among prediabetics.

**Table S2.** Sensitive analysis in excluding some concerns and high bias studies on T2DM incidence.

|  | RR(95%CI) | *I*^2^ | P |
| --- | --- | --- | --- |
| All studies | | | |
| Face-to-face intervention | 0.54(0.47,0.63) | 43% | ＜.001 |
| Digital health intervention | 0.88(0.77,1.01) | .6% | .06 |
| Blended intervention | 0.63(0.49,0.81) | .01% | ＜.001 |
| Excluding high-bias | | | |
| Face-to-face intervention | 0.55(0.45,0.68) | 61% | ＜.001 |
| Digital health intervention | 0.84(0.69,1.03) | 29% | .09 |
| Blended intervention | 0.69(0.49,0.97) | 0.0% | .03 |
| Excluding some concerns and high-bias | | | |
| Face-to-face intervention | 0.62(0.48,0.78) | 60% | ＜.001 |
| Digital health intervention | 0.85(0.68,1.06) | 47% | .16 |
